# Current Techniques of Gene Editing in Pigs for Xenotransplantation

**DOI:** 10.3389/ti.2025.13807

**Published:** 2025-05-27

**Authors:** Cesare Galli

**Affiliations:** Avantea and Fondazione Avantea Onlus, Cremona, Italy

**Keywords:** pig, genetic engineering, CRISPR-Cas9, somatic cell nuclear transfer, xenotransplantation

## Abstract

Shortage of human organs for transplantation has created a demand for alternative solutions of which xenotransplantation is amongst the most promising one in the short term. However, the immune reaction following transplantation of a pig organ is greater than the one elicited during allotransplantation. Genetic engineering of the pig is required so that pig organs or tissues are made less immunogenic to humans by eliminating some antigens and by expressing human proteins that can reduce the damage by the host immune system. To generate founder animals with the desired mutations genetic engineering of somatic cells with multiplexed mutations combined with somatic cell nuclear transfer (SCNT) is the best solution with the technology available today. Safety concerns include potential zoonosis, primarily porcine endogenous retroviruses (PERVs). Ethical considerations might arise from the use animals involved in research. Genome editing techniques based CRISPR-Cas9, have greatly facilitated the modification of pig’s genome to address coagulation and inflammation issues, to mention just a few, arising after the pig organ is transplanted into a human. However, further research is needed to ensure safety and efficacy of the genome edits introduced in the pig genome are compatible with the health and welfare of the pigs.

## Introduction

For many years, organ transplantation has been a critical life-saving option for individuals with end-stage organ failure. However, the gap between the need for organs and the available supply remains huge, resulting to long waiting lists, extended suffering, and preventable deaths. Xenotransplantation, which involves using organs from genetically modified animals, particularly pigs, offers a promising solution to this dilemma. Pigs are considered ideal donors due to their biological similarities to humans, compatibility in organ size, ease of breeding, genetic modification [1, 2], and the availability of gene editing tools [3, 4]. Additionally, pigs reproduce efficiently and have short generational intervals. Other livestock species are also being utilized as sources of biological materials for xenotransplantation or as bioreactors in biomedical applications [5–7]. Cattle, for example, are used to produce bioprosthetic heart valves from their pericardia [8]. Multiple immunological challenges have been identified (Table 1) and are being addressed through both genetic engineering and clinical immunosuppression protocols. Ensuring the safety of xenotransplantation is crucial, with particular concerns about the potential transmission of diseases, such as porcine endogenous retroviruses (PERVs) [9] and porcine cytomegalovirus (PCMV) [10]. Nevertheless, no cases of PERV transmission to humans following tissue xenotransplantation have been reported to date [11–13] and this threat has set back the field for more than 20 years. Recently, genetically modified pig organs have been successfully transplanted into brain-dead human patients [15, 16], and for the first time, into living patients under compassionate use [17]. Xenotransplantation also raises ethical questions concerning the welfare of animals used in research and the broader impact on animal populations both on the pig side but also on the use of NHPs for pre-clinical studies. The development of somatic cell nuclear transfer (SCNT) [18] and advancements in genome editing tools like CRISPR-Cas9 have revolutionized the field, leading to rapid progress in the genetic engineering of pigs for transplantation purposes [19, 20]. Scientists aim to modify pig genomes to increase compatibility with human recipients, minimize organ rejection, and reduce the risk of disease transmission. Although genome editing for xenotransplantation remains in the research phase, another emerging area of exploration involves creating pig-human chimeric organs, which presents significant scientific and ethical challenges [21]. This approach, again based on genome editing and SCNT, entails generating defective pig embryos for a specific organ, followed by combining them with human pluripotent stem cells (PSCs) to allow the PSCs to develop the targeted organ [22, 23], however at present it is not possible to prevent human PSCc to colonize other organs of the pig fetus. This review will discuss the various steps and challenges involved in generating viable animals, from selecting the target gene to cloning and animal birth.

**TABLE 1:** Immunological barriers to xenotransplantation that can be abrogated through Genetic Engineering (adapted from Perota and Galli, 2016) [14].

Problem	Possible cause	Possible solution
Hyperacute rejection (HAR)	Pre-formed antibodies against Galactose α1-,3-galactose and other non-Gal antigens (Neu5Gc); activation of the complement cascade	KO of α1-3 galctosyltransferase, CMAH, B4GALNT2, iGb3S and other non-Gal antigens Expression of hCRP (CD55, CD46, CD59)
Acute humoral xenograft rejection (AHXR)	*De novo* antibodies against Galactose α1-,3-galactose and other non-Gal antigens (Neu5Gc); activation of the complement cascade. Endothelial cell activation; Thrombotic microangiopathy Consumptive coagulopathy	hTBM, hEPCR, hA20, TFPI, CD39,HMOX1
Immune cell-mediated rejection (ICMR)	NK and T-cell activation	hTRAIL, CTLA4Ig, HLA-E, huβ2m, CD47, SLA class I
Instant Blood-Mediated Inflammatory Reaction (IBMIR)	Surface proteins, complement mediated, innate immunity, platelets and leucocytes activation	All of the above genetic modifications

KO, Knock Out; Neu5Gc, N-Glycolylneuraminic acid; CMAH, CMP-N-acetylneuraminic acid hydroxylase; B4GALNT2, Beta-1,4-N-Acetyl-Galactosaminyl Transferase 2; iGb3S, isogloboside 3; hCRP, human complement regulatory proteins; hEPCR, human endothelial protein C receptor; TFPI, tissue factor pathway inhibitor; TRAIL, human tumor necrosis factor related apoptosis inducing ligand.

## Genetic Engineering (GE) of Somatic Cells

In the past 2 decades, programmable nucleases have revolutionized genome editing, enabling precise alterations of the genetic code [19, 24–26]. Among these (see Table 2), CRISPR-Cas9 has become the most popular due to its simplicity, versatility, and low cost [27]. As the technology advances, more variants of CRISPR/Cas9 are expected to emerge. Effective application of these tools requires accurate DNA sequencing, along with software to assist in nuclease design, target site selection, and experimental validation, minimizing unintended effects known as off-target mutations [28–30]. SNPs present in different breeds or individuals might make inefficient the genome editing as the target site will not be recognized therefore it is important to sequence the target pig line to be used. These nucleases have been successfully used for gene editing across various species, including livestock, for both agricultural [31, 32] and biomedical [33, 34] purposes. In the biomedical field, pigs have long been a focus of genetic modification, particularly for xenotransplantation research. Typically, genetic editing in pigs target one [35, 36] or more specific loci, especially when inactivating endogenous genes through knockout (KO) methods. A well-known example is the genetic inactivation of the enzyme (GGTA1) responsible for the expression of the galactose α 1-3 galactose epitope (α-Gal) responsible for hyperacute rejection since is widely distributed on animal cell surfaces [37], followed by the KO of the enzyme CMAH, which is involved in producing the Neu5Gc antigen [36, 38–40] that is responsible for antibody mediated rejection. More recently, the simultaneous KO of GGTA1, CMAH, and B4GalNT2 (beta-1,4-N-acetyl-galactosaminyltransferase 2, also induces an antibody mediated response in humans) has been achieved [40, 41] and this genetic background is considered a basic requirement on where to build further gene edits. CRISPR-Cas9 has also been used to efficiently create multiple mutations in one round, for example, targeting three xenoantigens mentioned above simultaneously [40]. Another advancement in the CRISPR system involves the use of cytosine base editors (CBE), which can convert C to T without causing double-strand breaks (DSBs). This approach is used to silence endogenous genes by inducing nonsense mutations, offering a safer alternative to traditional methods like ZFNs or Cas9 [42, 43]. Since these modifications are made to cells cultured *in vitro*, researchers have ample opportunity to select cell clones with the precise mutation before using SCNT to generate the animals with the desired genotype. A simple and direct injection of CRISPR/Cas9 into zygotes can also produce genetically engineered animals [5, 44], this method though is less efficient when multiplexed genome editing is required and the risk of mosaicism, timepoint of microinjection is crucial to avoid mosaicism. Mosaic animals, which result from genetic editing occurring at later stages of embryonic development (cleavage stage), may not carry the desired mutations in all cells including germ cells and as a consequence do not transmit them to their offspring [45]. This risk is particularly concerning in livestock species with long generation intervals, making SCNT from validated cell clones a more reliable method ensuring that all the animals are carrying the exact mutation and will transmit to their progeny despite its low efficiency. In xenotransplantation, certain “safe harbor' loci can be targeted for KO of xenoantigens like GGTA1 [46] or CMAH, allowing for single-copy gene integration without disrupting other genes and at the same time ensuring expression of the transgene introduced. Additionally, specific solutions [47] could be used to address potential lethal effects of certain transgenes during embryonic development or early after birth. More advanced technologies may, in the future, help further control gene functionality. For example, if a transgene requires tissue-specific expression, such as in endothelial cells [48] or in insulin-producing cells [49], this can help minimize side effects of overexpression in all tissues that might impact homeostasis of the animal and ensure that genetic engineering (GE) remains compatible with the animal’s survival. Another method to control transgene expression is the use of inducible promoters, that can be activated by administering to the pregnant sows or after birth of the piglets, substances like tetracycline [50] or doxycycline [47]. This allows transgene expression to be switched on when needed, either during the animal’s life or after the organ is transplanted into the patient. Disadvantage of on-systems in the patient is a potential lifelong necessary administration of antibiotics. A third approach involves RNA interference (RNAi) technology, which has been used to reduce the expression of porcine endogenous retroviruses (PERVs) [51, 52], as they exist in multiple copies within the porcine genome, or to downregulate the expression of pig Tissue Factor [53], where a full gene knockout would be lethal. In such cases, small interfering RNA (siRNA) is highly effective, capable of reducing gene expression by 95% or more, though it does not completely eliminate the gene’s activity. One long-term issue with using commercially bred pigs for xenotransplantation is the continued growth of the organs after transplantation, potentially causing complications for the recipient [54]. This issue is relevant mainly for heart while kidney, for example, being in the abdominal cavity can tolerate the growth. One approach attempted to reduce size of the pig is to genome edit farm pigs to knock out the growth hormone (GH) receptor. However, with this size reduction, the pigs grow up to 60% of their normal size, also leads to unwanted health issues in the pigs since this mutation is responsible of a genetic disease making the breeding of these pigs not sustainable in the long term [55, 56]. Possible solutions already in development include using smaller breeds [57] or minipigs [58].

**TABLE 2:** Comparison of different programmable nuclease platforms used in livestock genome editing (adapted from [24] with permission from the Publisher).

	Zinc finger nuclease	TALEN	CRISPR/Cas9
Recognition site	Typically 9–18 bp per ZFN monomer, 18–36 bp per ZFN pair	Typically 14–20 bp per TALEN monomer, 28–40 bp per TALEN pair	22 bp (20-bp guide sequence + 2-bp protospacer adjacent motif (PAM) for *Streptococcus pyogenes* Cas9); up to 44 bp for double nicking
Specificity	Small number of positional mismatches tolerated	Small number of positional mismatches tolerated	Positional and multiple consecutive mismatches tolerated
Targeting constraints	Difficult to target non-G-rich sequences	5 targeted base must be a T for each TALEN monomer	Targeted sequence must precede a PAM
Ease of engineering	Difficult; may require substantial protein engineering	Moderate; requires complex molecular cloning methods	Easily re-targeted using standard cloning procedures and oligo synthesis
Immunogenicity	Likely low, as zinc fingers are based on human protein scaffold; FokI is derived from bacteria and may be immunogenic	Unknown; protein derived from *Xanthamonas* sp.	Unknown; protein derived from various bacterial species
Ease of *ex vivo* delivery	Relatively easy through methods such as electroporation and viral transduction	Relatively easy through methods such as electroporation and viral transduction	Relatively easy through methods such as electroporation and viral transduction
Ease of *in vivo* delivery	Relatively easy as small size of ZFN expression cassettes allows use in a variety of viral vectors	Difficult due to the large size of each TALEN and repetitive nature of DNA encoding TALENs, leading to unwanted recombination events when packaged into lentiviral vectors	Moderate: the commonly used Cas9 from *S. pyogenes* is large and may impose packaging problems for viral vectors such as AAV, but smaller orthologs exist
Ease of multiplexing	Low	Low	High

## From Genome Edited Cells to Animals

### Selecting the Cell Line

The choice of the cell line is a crucial factor in the success of Somatic Cell Nuclear Transfer (SCNT) embryo production and remains one of the key variables that can determine success or failure. Factors such as culture conditions, cell doubling rates, and oxygen levels [59] can influence which cell populations or sub-populations emerge during *in vitro* culture, affecting chromatin status and, most importantly, the ability of cells to be reprogrammed following nuclear transfer. Identifying cell lines with high SCNT efficiency can produce outstanding results, such as 10% livebirths on average being 2%–5% on the number of embryos transferred, whereas others may lead to significant failures [60].

For cloning purposes, skin fibroblasts from biopsies are the most used cell type when the genotype or phenotype of the donor animal is already known and must be perpetuated. However, when this is not the case, fetal fibroblasts are typically preferred, especially for genetic engineering (GE) applications. Some investigators also prefer to harvest early stage fetuses of 25–30 days of gestation [61, 62] if new edits must be added to that genetic background. While many studies debate the most efficient cell types for pig cloning [63, 64], these recommendations may sometimes conflict with the specific requirements of certain projects. In general, GE of the cell line used for SCNT does not significantly reduce its effectiveness in producing viable offspring, though a slight decrease in efficiency has been noted in gene knockout (KO) experiments [63, 64].

All cell lines can be cryopreserved at early passages before genetic modification, ensuring that the same cell lines can be reused in multiple rounds of nuclear transfer, thus controlling a critical variable in the SCNT process.

### Embryo Production

**FIGURE 1:**
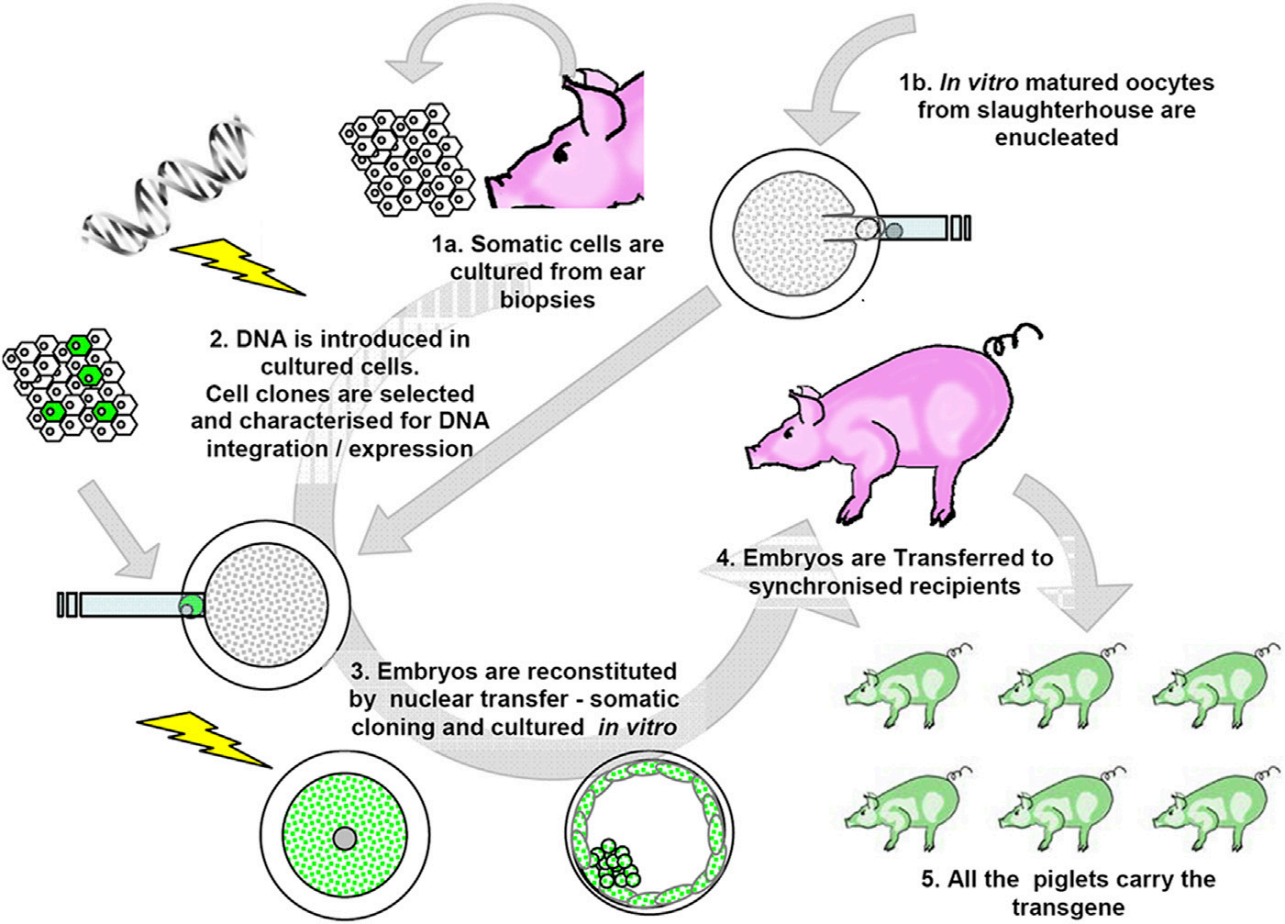
Transgenic production by SCNT. The transgene is transferred into the genome of cultured fibroblasts. Transgenic cell clones are isolated and characterized. This first step is relatively inexpensive. A more accurate prediction of the transgene expression is possible. Next, embryos are reconstituted by SCNT, cultured, and transferred in synchronized sows. Although the viability of cloned embryos is variable but usually poor, all of the resulting newborns carry the gene edits of the donor cell [66].

Over the years, the basic principles of cloning through nuclear transfer in livestock have remained consistent with the methods pioneered by Willadsen [65] and later adapted for somatic cells [18]. The process begins with the preparation of a matured enucleated oocyte, in which the metaphase plate together with the polar body is removed through micromanipulation. Next, a nucleus from a somatic cell carrying the desired genetic modifications is transferred into the enucleated oocyte by cell fusion by positioning the somatic cell into the perivitelline space or by adhering it with phytohemagglutinin in the case of zone free SCNT. Finally, the oocyte is activated either chemically or electrically to resume the cell cycle. The resulting embryos are then either transferred at the one-cell stage to the oviducts of synchronized recipient animals or cultured to the blastocyst stage before being transferred to the uterus of the recipient gilt (see Figure 1) [66]. A large number of metaphase II oocytes, necessary for embryo production, can be sourced inexpensively from slaughterhouses, adhering to the 3R principles (Replacement, Reduction, and Refinement). Procedures for oocyte maturation and embryo culture are well established in pigs [67] and are applied similarly in SCNT. However, micromanipulation remains a bottleneck in the process, requiring specialized equipment and skilled embryologists, as it is labor-intensive. Visualization of the metaphase plate is usually achieved using Hoechst staining and UV light, as livestock oocytes are rich in lipids, making them darker than those of mice or humans. Despite their normal morphology, SCNT embryos in the pre-implantation stage have reduced potential to develop to term [68]. This limitation is tied to the “black box' of cellular reprogramming, how the nucleus of the donor cell is reset to support normal embryo development. Currently, this process remains inefficient and is still poorly understood [69, 70] in livestock species, although some progress has been made in mice [71]. In mice, the use of Trichostatin A (TSA), a histone deacetylase inhibitor, during the early hours of culture after nuclear transfer has shown significant improvements in live birth rates by promoting chromatin demethylation and enhancing reprogramming [72]. Similar strategies, involving various demethylating agents, have shown promising results in pigs in some laboratories [73].

### Pregnancy

The ability of SCNT embryos to establish pregnancies is generally lower than that of embryos created through fertilization. This discrepancy has economic implications, as it increases the cost of maintaining recipient animals that are either not pregnant or that experience pregnancy loss. In pigs, this issue can be partially mitigated by transferring a large number of embryos, as SCNT embryo production is typically not a limiting factor. Pigs can tolerate the transfer of over 100 embryos, adjusting naturally through physiological reabsorption of excess embryos. Another challenge with SCNT pregnancies is that they often have a prolonged gestation period, frequently necessitating induced parturition or cesarean sections because of the presence of a small number of fetuses or fetuses of small size [74].

### Offspring

The success rate of SCNT in terms of development to term can reach up to 16% [75], depending on whether the rate is calculated based on the number of reconstructed embryos or transferred blastocysts. However, various factors, such as pig breed and the cell line used for genetic modification, can affect this success rate, making comparisons difficult [64, 76]. SCNT offspring are more fragile at birth and have higher stillborn and perinatal mortality rates. To improve their survival, special attention is required during farrowing and neonatal care in the first week of life. Once they survive the critical first days or weeks, cloned animals generally live normal lives, are fertile, and do not pass on any phenotypical abnormalities to their offspring [77–80]. An important consideration for the broader application of this technology is the impact of genome editing on the viability of the animal and its ability to breed naturally. It’s crucial to determine how many genome edits are necessary and compatible with maintaining the animal’s homeostasis [81, 82], as well as whether the inserted transgenes are expressed at the desired levels. This requires thorough genotyping and phenotyping of newborn animals. A systematic approach might be necessary to assess each genetic modification before proceeding further. This should also be confirmed in the F1 generation (F0 are considered the founder animals) to be sure that the expression of the transgenes is maintained while breeding the animals by sexual reproduction. Once the pig line is established, to be sustainable from an economic point of view and for health and welfare reasons for the animals conventional breeding should be the priority. One example of potentially unnecessary genetic modification is the inactivation of all 64 copies of porcine endogenous retroviruses (PERVs) in a pig line [9], even though there has never been documented transmission of PERVs to humans in previous xenotransplantation experiments [11–13]. Additionally, PERVs may have an as-yet-undiscovered physiological role in the genome, which warrants further studies [83, 84].

## Conclusion

Remarkable progress has been made in the genetic engineering of pigs in general and specifically to produce organs suitable for transplantation to humans. This has lead to the first pig to human transplantation of heart in living patients under compassionate use [17, 85] and in 2024–2025 the xenotransplantation of kidneys into clinical patients [86] at the same time FDA has given approval for the first IND application to initiate clinical trials [87]. Gene-editing technologies, particularly CRISPR-Cas9, have been employed to modify pig genes associated with immune rejection, viral transmission, and compatibility issues, leading to the development of pigs with reduced immunological challenges and increased suitability for human recipients. Genome editing technologies are continuously developed to make them more effective and adaptable to the different need of gene editing while reducing potential side effects [88, 89]. However, one of the primary obstacles in xenotransplantation remains the immune response triggered by pig organs in humans, which results in organ rejection. Genetic engineering is focused on overcoming this barrier by altering or removing problematic genetic elements. Although substantial advancements have been achieved, further research is essential to ensure long-term graft success and avoid immune-driven rejection. The genetic modification of pigs also raises ethical questions, particularly regarding animal welfare and the broader implications of genome alterations in animals. It is vital to ensure the wellbeing of genetically modified pigs, guided by strict ethical standards and practices. As a highly regulated and complex field, xenotransplantation requires extensive preclinical research, safety evaluations, and approval from regulatory bodies before it can become a standard medical procedure. Clinical trials will be necessary to assess the safety and effectiveness of pig organ transplantation in humans and a number of non-human primates will also be required for that. In summary, genetically engineering pigs for xenotransplantation holds great promise in addressing the global organ shortage. While considerable strides have been made, more research is needed to overcome immunological challenges, reduce the risk of pathogen transmission, address ethical concerns, and meet regulatory and clinical requirements. Future scientific developments, combined with rigorous safety protocols and ethical considerations, will be pivotal in successfully translating pig genetic engineering into viable and safe xenotransplantation therapies while preserving the health and welfare of the animals involved.
